# Participant and caregiver perspectives on health feedback from a healthy lifestyle check

**DOI:** 10.1111/hex.13960

**Published:** 2024-01-12

**Authors:** Miranda D. Lee, Cervantée E. K. Wild, Ken J. Taiapa, Ngauru T. Rawiri, Victoria Egli, Sarah E. Maessen, Yvonne C. Anderson

**Affiliations:** ^1^ Department of Paediatrics Te Whatu Ora Taranaki New Plymouth Taranaki New Zealand; ^2^ Department of Paediatrics: Child and Youth Health University of Auckland Auckland New Zealand; ^3^ Nuffield Department of Primary Care Health Sciences University of Oxford Oxford UK; ^4^ Ngāti Porou, Rongowhakaata Tangata3 New Zealand; ^5^ Ngāti Mutunga, Ngāti Rāhiri o Te Ātiawa Ngai Tūhoe, Ngāti Ruapani mai Waikaremoana, Ngāti Pāhauwera (iwi) New Plymouth Taranaki New Zealand; ^6^ School of Nursing, Faculty of Medical and Health Sciences University of Auckland Auckland New Zealand; ^7^ Faculty of Health Sciences Curtin University Bentley Western Australia Australia; ^8^ Telethon Kids Institute Perth Children's Hospital Nedlands Western Australia Australia; ^9^ Child and Adolescent Community Health Child and Adolescent Health Service Perth Western Australia Australia

**Keywords:** child health services, consumer health information, health communication, qualitative research

## Abstract

**Introduction:**

The usual output following health consultations from paediatric services is a clinical letter to the referring professional or primary care provider, with a copy sent to the patient's caregiver. There is little research on how patients and caregivers perceive the letter content. We aimed to: first understand child, young people and caregiver experiences of and preferences for receiving a health feedback letter about the child/young person's health measures within a healthy lifestyle programme; and second to provide a set of recommendations for designing letters to children, young people and their families within a healthy lifestyle programme.

**Methods:**

This qualitative study, informed by Kaupapa Māori principles, included focus groups of children aged 5–11 years and young people aged 12–18 years who were participants in a healthy lifestyle programme in Taranaki, Aotearoa New Zealand and of their respective caregivers (total *n* = 47). Discussions were audio‐recorded, transcribed and analysed using thematic analysis.

**Findings:**

Key themes were identified: letters sometimes acted as ‘discourses of disempowerment’—some participants experienced a lack of safety, depersonalisation with medical jargon and ‘feeling like a number’. Participants described the need for acknowledgement and affirmation in written communication—health feedback should include validation, choice regarding content, respectful tone and a strengths‐based approach to health messages.

**Interpretation:**

Letters to referrers, copied to families, can be perceived as disempowering, and participant and caregiver perspectives of content should be considered. This study challenges conventional practice in communicating health feedback with broader implications for written communication in healthcare. We propose separate letters aimed at the child/young person and their caregiver that offer choice in the information they receive. The administrative burden of multiple letters can be mitigated by advances in digital health.

**Patient Contribution:**

This study originated in response to feedback from service users that current health feedback was not meeting their needs or expectations. Patient perspectives, especially from children, are rarely considered in the generation of clinic letters from health professionals. Participants were child participants in the community‐based clinical service and their caregivers, and care was taken to represent the demographic backgrounds of service users. Collection and interpretation of Māori data were led by researchers who were local community members to ensure prioritisation and preservation of participant voice. Where possible, results are illustrated in the text by direct quotes from participants, whose identities are protected with a pseudonym.

## 1 INTRODUCTION

There are numerous instances in a healthcare journey where patients experience difficulties receiving feedback and information. Potential difficulties navigating information or accessing services may be encountered at any stage of the healthcare journey, from referral and assessment to feedback of health information.^1^, ^2^, ^3^ This is particularly evident in sensitive areas of healthcare such as the support of children and young people affected by overweight and obesity. The World Health Organization recommends family‐based multidisciplinary programmes to address childhood obesity, but these are not widely available, accessible or acceptable in many jurisdictions, including Aotearoa New Zealand (NZ).^2^, ^4^ Māori health researchers, among others, have identified challenges in communicating the issues of obesity without generating ‘fat‐shaming’ or ‘victim‐blaming’ experiences for those affected,^5^, ^6^, ^7^ especially when support services are limited. Whānau Pakari, based in Taranaki, NZ, was designed to ensure accessibility and appropriateness of a healthy lifestyle service, and to minimise barriers to engagement.^1^ The family‐focused, community‐based assessment and intervention healthy lifestyle programme follows international best practices to support healthy lifestyle changes for children and adolescents affected by obesity or weight‐related comorbidities.^8^ Whānau Pakari includes a comprehensive home‐based weight‐related assessment, with weekly healthy lifestyle group sessions held in the community.^1^, ^8^

If done appropriately, communicating health feedback fulfils professional obligations to inform patients about their health and treatment options, and provides a record to ensure consistency between doctor and patient understanding of clinical interactions.^9^ Following health consultations from paediatric services, usual practice involves a clinical letter to the referring professional, with a copy of this letter sent to the patient's caregiver. Typically, these letters contain medical information relevant to a clinician but may not include the information deemed most relevant to children, young people and their families. In Whānau Pakari, standard health information conveyed in clinical letters includes body mass index standard deviation score (BMI SDS), blood pressure, results of relevant investigations and information relating to dietary behaviour, physical activity and wellbeing. There is increasing recognition of the importance of clinical letters to communicate health information to patients; however, they often fall short of their potential in current practice.^10^ To our knowledge, no research to date has asked children and young people for their opinions and preferences for receiving health information in a letter after using a clinical service.

This study aimed to understand children's, young people's and caregivers' perceptions of health feedback from the Whānau Pakari programme and the wider healthcare system. Second, it aimed to explore novel ways of providing feedback in letters for Whānau Pakari participants and beyond.

## 2 METHODS

### 2.1 Study design

This qualitative study was informed by a critical approach to qualitative research and Kaupapa Māori principles. Kaupapa Māori research methods refer to design, data collection and analysis, which incorporate a Māori world view, philosophy and cultural principles.^9^, ^11^ The lead researcher was non‐Māori but was supported by Māori facilitators at the focus groups with senior Māori researcher oversight. This study aimed to recruit equal numbers of Māori and non‐Māori participants to ensure recommendations for acceptability and appropriateness were relevant for families who identified as Māori within the programme.

Ethical approval for this study was granted by the Auckland Health Research Ethics Committee, University of Auckland, NZ (reference AH22704). Ethics for this study did not allow for the publication of artwork/handwriting as these were deemed identifiable. Explicit consent for publishing participants' drawings was therefore not obtained.

### 2.2 Clinical context and study participants

Children or young people are typically referred to the Whānau Pakari programme by a primary healthcare provider, but anyone in the community may make a referral. On entering the programme, weight‐related assessments are undertaken in the family home by a trained healthy lifestyle coordinator. These consist of questionnaires surrounding eating behaviour, exercise and well‐being alongside health measurements, including BMI SDS and blood pressure.^8^ Recommendations and goal setting are offered with the opportunity to attend weekly activity sessions run by a dietitian, psychologist and physical activity coordinator. A letter to the referrer is generated from the assessments using the programme database, with a copy sent to the family.

Inclusion criteria for this study were families and children/young people participating in Whānau Pakari between 1 January 2019 and 30 September 2021 who had received a health feedback letter. Detailed criteria for participation in Whānau Pakari are described elsewhere.^8^ Programme staff made initial contact with families via telephone or face‐to‐face at Whānau Pakari weekly group meetings. Interested families were contacted by researcher ML via telephone to provide further information, including discussion of participant information forms and potential focus group dates. Families had the opportunity to accept or decline to participate at any stage without providing a reason.

Written informed consent was obtained from all participants aged 16 years or older. All participants younger than 16 years completed assent forms, with written consent from their caregivers. Demographic data were collected following NZ protocols for ethnicity data collection (Table 1).^12^

**Table 1:** Participant demographics.

	Children (5–11 years)	Young people (12–18 years)	Caregivers	Total
*n*	16	9	22	47
Female a	8 (50%)	4 (44%)	19 (86%)	31 (66%)
Ethnicity^b^, ^c^				
Māori	9 (38%)	4 (31%)	8 (29%)	21 (32%)
New Zealand European	13 (54%)	7 (54%)	18 (64%)	38 (59%)
Pacific Peoples	2 (8%)	1 (8%)	1 (4%)	4 (6%)
Asian	0	1 (8%)	1 (4%)	2 (3%)

^a^ One participant identified their gender as ‘preferred not to disclose’. They have been aggregated here to avoid identification.

^b^ Total ethnicity output (people are counted more than once in each ethnic group if more than one ethnicity reported).

^c^ Ethnicity data collected according to Ministry of Health protocols (12).

A total of eight focus groups lasting approximately 60–90 min took place over four separate dates in September/October 2021, in the Taranaki region. Participants were sampled to represent those in North and South Taranaki to include those from different socioeconomic and geographical areas. There were two groups of children (aged 5–11 years), two groups of young people (aged 12–18 years), and two groups each of their respective caregivers (totalling eight focus groups). Other family members were invited to attend, consistent with the family‐centred approach of the research. Table S1 outlines the focus groups and their respective participant numbers, associated research facilitators and locations. All focus groups were audio‐recorded and began with whakawhanaungatanga (relationship‐building) activities, including shared kai (food).

A total of 79 families with 84 eligible children/young people were approached to take part in the study. Twenty‐five families could not be contacted, 11 families declined to participate, and 43 interested families agreed to attend the focus groups. Of the 27 children/young people and 23 caregivers who attended the focus group sessions, one child, plus one young person and their accompanying caregiver, declined to participate on the day. Demographic information for the 47 participants who took part in the focus groups is detailed in Table 1, and participants have been given pseudonyms to protect their identities.

### 2.3 Procedures

Focus group prompts asked about participants' experiences with Whānau Pakari and how they would like staff to communicate with them about their health in a letter (File S1). An example existing letter to the referrer (or stated lead health professional) was shown to participants for their reflection and opinions. Participants worked together to consider what an exemplar letter from the Whānau Pakari clinical team to the child, young person and their caregiver would look like using the materials in the creative kit provided (coloured pens and paper).

A koha (gift, gesture of thanks) in the form of sports/book and fuel vouchers was offered to all participants of the study.

### 2.4 Analysis

Audio recordings were independently transcribed, and creative writing/drawings were photographed and entered into NVivo Release 1.6.1 (QSR International, January 2022) for analysis. Inductive thematic analysis was used to code data (transcripts, creative writing and drawings) using Braun and Clark's^13^ methods, led by researcher M. L., with supervision from researchers C. W. and Y. A. After familiarising themselves with the data, M. L. coded transcripts and photographs semantically, along with some interpretive codes. A mind‐mapping process was used to visualise relationships between codes and to generate initial themes. The final themes were discussed and sense‐checked within the wider research team. A set of recommendations for clinic letter design were also generated by the team as an iterative process throughout analysis and writing, grounded in the focus group data, supported by children's drawings and young people/caregivers' example letters.

### 2.5 Role of the funding source

No funders had any involvement in study design; in the collection, analysis or interpretation of data; in the writing of the report; nor in the decision to submit the paper for publication.

## 3 RESULTS

This analysis identified both anticipated and unforeseen insights in relation to health feedback. First, we describe two key themes constructed from the data that provide deeper insights into participants' perspectives of their health letter: (1) discourses of disempowerment and (2) the importance of acknowledgement and affirmation. Second, we describe a set of recommendations on what their health letter should look like, drawn from insights of the child, young person and family perspectives.

### 3.1 Theme 1: Discourses of disempowerment

This theme encompasses the discussions and narratives surrounding the lack of power and agency experienced by study participants. Some participants shared experiences of feeling disempowered by their feedback letters, expressing sentiments of being reduced to a statistic, feeling a lack of safety, being overlooked, patronised and confronted with confusing medical terminology.

Participants described how the existing letter to the referring health professional felt disempowering. It illustrated how confronting the written word can be, even if unintentional, and how unsafe this may feel to a person and their family. Some caregivers expressed feeling as though they were inadvertently treated like a number, which felt like a lack of compassion.

One mother described a lack of safety when reading about their child's health in the letter and the subsequent effect on wellbeing.

…it's interesting how when you get that letter, how unsafe you can feel just by receiving this, and you … start to feel like you're a number, you're being added to statistics, and you're less than what you thought you were at the beginning. So, health and well‐being really are undermined if you don't have safety in your home and community and within the system if you're marginalised. (Marama, Mother, Māori)

Participants described feeling unseen/unheard regarding their existing efforts to achieve and maintain health goals, which were not validated in the letter:

…we also want to see that they see and hear us in our context and that they represent that in the kōrero [conversation]. You guys are playing this sport and that sport, it just means you do see us. (Rachel, Mother, Māori, Pasifika, NZ European)

The traditional style of outpatient clinic letters can be clinical and lack the humanistic qualities of viewing each person as an individual. When their efforts are unseen, the individual and their family can feel unimportant, which may ultimately have an adverse effect on engagement with the health system.

Some participants reported that they felt their voices and needs were not being reflected by the ‘generic’ healthy eating/exercise recommendations in the current letter format, which felt patronising.

And angry, and this is all shit!!! Look at this, eat more this, do, it's crap … we're already doing that and … the exercise one was … oh, do some more exercise. How can you do more when you're already doing five days, six days a week? (Marama, Mother, Māori)

Patronising behaviour can exacerbate any power imbalance in interactions and can undermine confidence, autonomy and agency, thus resulting in disempowerment. From an adolescent perspective, patronising behaviour was reported as annoying and led to disengagement.

A teenager, reflecting upon his current generation being born into an era of electronic devices, found it patronising when adults recommended limiting screen time. They considered this ‘insincere’ and an ‘unrealistic expectation’, ‘…when the same people you get that advice from go home and watch television … for more than an hour a day’ (Richard, aged 12–18 years, NZ European).

Almost universal amongst focus groups were difficulties understanding medical jargon in the letter. When health professionals use medical jargon without full explanation, it can lead to a lack of understanding of their health measures, as well as reduced agency and disempowerment. Participants disliked results being displayed with numbers and scientific measurements (e.g., millimoles), and many did not know the results' significance for their child's health.

It's like it was written in a different language half the time. (Michael, Father, Māori/NZ European)

Overall, caregivers expressed a preference for smaller paragraphs, fewer words, bullet points and ‘words that everyone can understand’ (young people aged 12–18 years, group work creative writing extract). A participant described how they did not understand terms like glycated haemoglobin (HbA1c), suggesting that some medical jargon in patient letters inhibits participants' understanding of their health, impedes agency and is disempowering. Caregivers were frustrated with wording that placed the burden on the family to understand the health information. One described looking up medical words on the internet while unsure if the source was reputable. It was clear that medical jargon included in patient letters was designed for health professional communication, not the child/young person and their family.

The paper, the postage, the effort of sending this is useless unless it is more user‐friendly for the layperson … because it's probably all going in the bin, or worse, the work is put on us where we are on the internet for an hour like, what does this mean? (Beatrice, Mother, NZ European)

Participants described how the letter felt like a formal document, which can be intimidating.

It … looks like what you get after an accident, like … the ACC [Accident Compensation Corporation] form. (Suzy, Mother, Māori, NZ European)

### 3.2 Theme 2: The importance of acknowledgement and affirmation

Participants described the need for acknowledgement and affirmation in written communication in their letter. They felt that letters should be respectful, include validation, choice in what feedback was included, and ensure a strengths‐based positive approach. Participants expressed how they had affirming conversations during their face‐to‐face assessments with the healthy lifestyle coordinator(s), but the letter they received did not reflect this experience. One participant shared, ‘it's like I need to have some positive affirmations … I think getting the letter afterwards you're kind of like, “that's not how I thought it seemed”’ (Zoe, Mother, NZ European).

In contrast, participants described how receiving a personalised letter with health feedback could be an enabling and validating experience, saying, ‘[*it would be*] helpful when it's personalised’ (Suzy, Mother, NZ European/Māori). Participants felt they should be given choice of which clinical parameters were included in their letter, according to what mattered most to them as a family.

Caregivers described the importance of acknowledging the child and caregiver's wider journey with respect to complex issues or a family crisis, limiting their capacity to implement the programme's advice.

At least three of us were going through something quite dramatic at the time, which may or may not have impacted with how engaged we could have been. (Zoe, Mother, NZ European)

However, some participants would not want such information written in their letter, suggesting that choice is important.

Caregivers, children and young people described the need for a strengths‐based approach, with feedback providing encouragement and positive messaging rather than negative language. One of the young peoples' improved letters, produced in one of the focus groups, included the desire for ‘encouragement that I can do it. That it does take time to make a change’ (Creative writing extract, group work, author(s) unknown, aged 12–18 years). Children wanted to have health information communicated ‘respectfully’ and ‘calmly’. Especially within a Māori cultural context, respect is important.

Younger children wanted to know how they were doing, if they were ‘sturdy enough’, and to learn about new activities and healthy foods. However, older children indicated a preference for being treated like adults, without information hidden from them. One teen wanted ‘brutal honesty’ in communication about their health. This included not treating them like a child: ‘…don't try and comfort me because if you treat me like an adult then, that's how I want to be treated … I'm not some eight‐year‐old, I'm seventeen years, you know’ (Richard, aged 12–18 years, NZ European). This demonstrates the importance of tailoring clinic letters for different age groups and providing choice in letter content.

Young people's group creative writing extracts described empowering and less definitive language such as the word ‘maybe’ to demonstrate recommendations as positive suggestions rather than orders.

Maybe you could do more exercise, and don't forget the portions. (Creative writing extract, group work, author(s) unknown, aged 12–18 years)

Similarly, another writing extract suggested a desire for positive affirmation.

You should make some goals on healthier eating and focus on achieving these little by little. Adding small bits of exercise at the start and building would be helpful. Thank you for taking in this information. Never give up. (Creative writing letter design group work, aged 12–18 years)

It was repeatedly highlighted that acknowledging existing efforts to achieve healthy lifestyle change was important. Caregivers wanted more positive affirmations of what the children were doing well.

Focus on the things that are being achieved … so you can't run around the block yet, ‘that's terrible’ or you can go ‘oh, you are now running half a block more than you were running three weeks ago, that's progress’…. (Elizabeth, Mother, NZ European)

**Figure 1:**
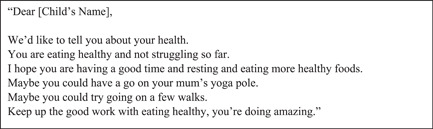
Letter designed by child, group work creative writing extract, aged 5–11 years, author(s) unknown.

The sample of children's group creative writing in Figure 1 demonstrates strengths‐based, encouraging language, using ‘maybe’, absence of medical jargon, and positive validation, and demonstrates acknowledgement and affirmations. Aesthetically, children wanted their letters to be visually engaging with pictures of their drawings, animated characters, people and animals. Young people wanted letters with colourful fonts, illustrations and funny jokes.

Alongside letters to the referring health professional following an appointment, many participants confirmed that a letter to the caregiver and adapted versions for the child/young person could be provided. Using data from focus groups, the following key findings/recommendations for health professionals to use in their feedback letters were collated, as outlined in Table 2.

**Table 2:** Participant and caregiver recommendations for health feedback letters.

Recommendation	Key related theme	Supporting example/suggestions
Adopt an individualised, personal approach.	Discourses of disempowerment.	Ensure the family and child's names are included in the letter with a welcoming, kind and respectful tone (e.g., Figure 1). ‘[It would be] helpful when it's personalised.’(Suzy, Mother, Māori/NZ European)
Include holistic and strength‐based measures alongside conventional biomedical outcome measures.	The importance of acknowledgement and affirmation.	Medical health measures may be desired by some, and if used should have all medical jargon explained with the implications for the child. …‘the work is put on us where we are on the internet for an hour like, what does this mean?’ (Beatrice, Mother, NZ European)
Tailor the letter to the intended reader.	The importance of acknowledgement and affirmation.	A letter to the young child could have visual illustrations created by the child to assist them to connect the feedback to a previous interaction with the service. ‘It would be cool if they had any … pictures that kids draw, like when they are talking to our parents you can maybe be drawing or something, then they put it in the letter, and you're like ‘oh yeah now I remember—they were talking about my health and this is the picture I did’. (Heaven, female age 5–11 years, Māori/NZ European)
Use suggestive language rather than imperatives in recommendations for change/management plans.	Discourses of disempowerment. The importance of acknowledgement and affirmation.	Use positive suggestions rather than ‘orders’. ‘Maybe you could do more exercise, and don't forget the portions’. (Creative writing extract, 12–18 years group work, author(s) unknown)
Celebrate achievements and document goals.	The importance of acknowledgement and affirmation.	Include a section for goal‐setting and flexibility relating to order of information, and where possible, choice about letter content. ‘You're doing amazing, and keep up the good work’. (Creative writing extract, 5–11 years group work, author(s) unknown) ‘I think it's just goals of changing what you eat and making it a little healthier. You could start off little and progress from then on’. (Lily, female, Māori/NZ European/Pasifika age 12–18 years)

## 4 DISCUSSION

In this study, we found that existing feedback letters received by caregivers from a multidisciplinary healthy lifestyle programme could be perceived to be disempowering to participants. Such letters, which are addressed to the primary healthcare provider and copied to the patient or caregiver, are typical in wider medical practice. Our results demonstrate a need for change from healthcare practitioners writing a letter ‘about’ the patient to the referrer towards a letter ‘to’ the child/young person and their caregiver. We recommend letters be tailored to the recipient, with medical jargon fully explained and a choice in what health information is included. Overall, participants expressed preference for an empowering approach with strengths‐based, personalised, affirming language to support healthy lifestyle change. Making letters visually appealing and age‐appropriate was important to Māori and non‐Māori children and young people in our study.

We are not aware of any previous research including child and family perspectives on letters following a health service visit. However, parents who were notified that their child was an unhealthy weight after participation in a school‐based screening programme in England described feeling shocked, disgusted and judged by the letter they received.^14^ Though not directly comparable to a setting where weight issues have already been identified, these reactions are congruent with the feelings of disempowerment expressed in our focus groups, demonstrating the power that medical correspondence could have to enhance or undermine engagement with a clinical service. Navigating a complex health system to access care can be difficult for families in NZ, with negative past experiences identified as a key barrier to accessing or remaining engaged in health services.^5^, ^15^

For many who identify as Māori, a further barrier to accessing hospital services is a lack of health information, which, when available, is often poorly explained.^16^ The NZ Whānau Ora initiative^17^ and, more recently, the Whakamaua Māori Health Action Plan 2020–2025^18^ aim for empowered whānau [families] having ‘access to quality information, advice, resources, a sense of agency and self‐determination’. Perspectives on the existing feedback letter used in Whānau Pakari in the current study demonstrate that in the child health setting, this aspiration is yet to be realised. Some participants in our focus group described medical jargon not just as a barrier to understanding health feedback but also as a burden to decode.

A study in the United Kingdom used ‘enhanced’ health feedback letters to caregivers to improve the likelihood of using a weight management service, which was attributed to a better understanding of information about their child's health.^19^ Although the letter focused primarily on weight, which our study indicates is not helpful or relevant to all families, visual components were a key ‘enhancement’ used to simplify complex health concepts. Visually interesting letters were a preference for children and young people in the present study, but their potential to enhance understanding of health feedback for all family members warrants further investigation. In the health research space, Kearns et al.,^20^ had success in engaging Māori adults to participate with the use of comics, and Egli et al.^21^ used visual components to engage child participants and facilitate their understanding of study results. Incorporating children's own artwork into their letters has a further role in facilitating their connection with health interactions. Thus, visual elements in letters to families accommodate the preferences of children and may help both children and adults to be better empowered to understand and act on health feedback.

The United Nations Convention on the Rights of the Child emphasises the importance of considering children's views in matters that affect them, yet children's voices are conspicuously scarce in most areas of child health research.^22^ As far as we are aware, no previous research has sought children's views on written communications from health services. One study included child perspectives on preferences for in‐person discussions about weight, with both children and caregivers advocating a strengths‐based approach, and an emphasis on growth and health rather than weight and size.^23^ This sentiment was echoed in our focus groups, with participants noting the absence of this approach from clinic letters as a contrast from their in‐person experiences. Participants felt that an approach to health feedback that highlights achievements would be more engaging and validating than traditional feedback, which was perceived as focusing on negatives. Egli et al.'s^21^ advice for dissemination of health research to children highlights the importance of being adaptable, incorporating children's feedback and meeting children where they are at in the digital and physical world while being cognisant of the words used.

Recently, the Academy of Medical Royal Colleges recommended that letters be addressed to patients, with recommendations for clear communication and explanation of all medical jargon.^10^ Though uncommon in most clinical settings, this approach is typical in reports to families from clinical geneticists.^24^ Literature in the adult setting suggests that writing directly to the patient improves collaborative decision‐making, communication and patient understanding.^25^ In our study's context, involvement in letter writing by having choices in what health feedback is included and how it may improve recipients' agency for their own health. However, this must be balanced alongside medical council guidance recommending clinical investigations, and results should be conveyed to caregivers, children and young people.^26^ Further, research suggests that caution must be exercised as we move to more agentic models of healthcare, which risk impacting negatively on outcomes for Māori.^6^

The logistics of creating 2–3 separate letters from each patient encounter would be greatly simplified with digital technology to populate multiple letters with written and visual content. Feedback from Whānau Pakari participants indicates a willingness to embrace digital technology, as long as it augments rather than replaces face‐to‐face interactions.^27^

As a key strength, this study prioritises the voices of children and young people. However, these voices were acknowledged to be less prominent than caregivers overall. Observations from our study highlighted that young children showed little interest in providing feedback on the current style of their feedback letter, aligning with their developmental stage and typical childhood preferences. Hence verbal quotes from young children are limited in this study and theme. Young children's viewpoints are described in the recommendations. Very young children often have limited autonomy and verbal expression, which can contribute to a sense of disempowerment. Despite the relatively limited quantity of verbal data from young children in this study, their input remains highly important. A limitation was that Māori participants comprised 32% of the cohort, falling short of our aim of recruiting equal numbers of Māori and non‐Maori participants, but this was greater than the population prevalence (20% in Taranaki district health board region in 2018).^28^ NZ was in a COVID‐19 lockdown during September 2021, where gatherings of up to 100 people were allowed. There was a reticence to meet in group settings for many, and there remained a requirement for physical distancing within smaller spaces for example, meeting rooms. These factors may have affected recruitment numbers. Future research could beta‐test newly designed letters, incorporating feedback from children and families accessing different types of paediatric healthcare, including in hospital and community‐based healthcare services for children.

In conclusion, despite the strong commitment to child and family‐centred care within Whānau Pakari, this study highlighted that generic letters from the programme were often perceived as disempowering. With the use of digital technology, it will be possible to provide a feedback letter to the referring health professional, caregiver and child or young person, enabling the child and family to experience strengths‐based feedback to support healthy lifestyle change, with an element of choice as to how the feedback is presented. The wider applicability of these findings to improve how health information is provided to children within the broader health system warrants further exploration.

## AUTHOR CONTRIBUTIONS

**Miranda D. Lee**: Methodology; formal analysis; data curation; writing—original draft; investigation; funding acquisition; project administration. **Cervantée E. K. Wild**: Methodology; supervision; writing—review and editing. **Ken J. Taiapa**: Data curation; supervision; writing—review and editing. **Ngauru T. Rawiri**: Data curation; supervision. **Victoria Egli**: Methodology; writing—review and editing. **Sarah E. Maessen**: Writing—review and editing. **Yvonne C. Anderson**: Conceptualisation; methodology; funding acquisition; supervision; writing—review and editing.

## CONFLICT OF INTEREST STATEMENT

The authors declare no conflicts of interest.

## Supporting information

Supporting information.

Supporting information.

## Data Availability

Data from this study will not be made available, as the participants of this study did not give written consent for their data to be shared publicly.
