# Supplementary material for: Participant and caregiver perspectives on health feedback from a healthy lifestyle check
Source: Health Expect. 2024 Jan 12;27(1):e13960. doi: 10.1111/hex.13960 (PMC10785704; doi:10.1111/hex.13960)
Supplement: Supplementary file 1 — Supporting information. [file HEX-27-e13960-s001.docx]

**Participant and caregiver perspectives on health feedback from a healthy lifestyle check**

**Supplementary Table 1.** Participant number in each sub-group.

| Focus group | Group composition | Group size | Siblings | Researcher |
| --- | --- | --- | --- | --- |
| **1** | Child 5-11 years* | 10 | 6 children (3 sets of siblings) | KT  NR, ML |
| **2** | Adult caregivers of 5-11 years | 7 | - |  |
| **3** | Child 5-11 years | 7 | 2 children | KT  ML |
| **4** | Adult caregivers of 5-11 years | 6 | - |  |
| **5** | Young person 12-18 years | 5 | 0 | KT, ML  NR |
| **6** | Adult caregivers of 12-18 years | 5 | - |  |
| **7** | Young person 12-18 years | 3 | 0 | KT  ML |
| **8** | Adult caregivers of 12-18 years | 4 | - |  |

*One sibling in attendance age 12-18 years.

Adult caregivers included parent/grandparent or legal guardian. One caregiver attended without a young person.

Focus groups with child/young person ran concurrently with their respective caregivers at separate tables on the same date and venue with KL or NR providing Māori cultural facilitation.

Groups were mixed genders, apart from focus group 8 (all female adult caregivers)*.*
