# Supplementary material for: Participant and caregiver perspectives on health feedback from a healthy lifestyle check
Source: Health Expect. 2024 Jan 12;27(1):e13960. doi: 10.1111/hex.13960 (PMC10785704; doi:10.1111/hex.13960)
Supplement: Supplementary file 2 — Supporting information. [file HEX-27-e13960-s002.docx]

**Supplementary File 1.** Focus group questions.

**Focus Group Exercise 1: Informing the child** (20mins)

Think back to when you first started with Whānau Pakari.

Q 1. How did Whānau Pakari staff talk with you about your health?

Q 2. How would you have liked the Whānau Pakari team and other health professionals (people like doctors and nurses) to communicate with you (the child) about your health, and progress through your health journey?

Q 3. If you were put in charge of how the Whānau Pakari team communicates these things with other kids in the future how would you do it?

Using the materials in the creative kit, work together to create what you think would be the ideal way for the Whānau Pakari team to communicate health feedback to other kids, and how a letter about your health would look like.

**Example Exercise 2: Informing the Family** (10mins)

Think back to when you first started with Whānau Pakari. Using the materials in the kit work together as a family to create a way for Whānau Pakari to communicate to you as a family.

Q.1 What would you have liked the Whānau Pakari team to tell you about your whānau health?

Q.2 How would you have liked the Whānau Pakari team to inform you of these things?

Q.3 If you were put in charge of how the Whānau Pakari team communicates these things to other families in the future, how would you do it?

**Example Exercise 3: The Whānau Letter** (10mins)

Using the materials in the creative kit work together to create a letter from the Whānau Pakari team to your family. In this letter you should include what your health is like and about your healthy lifestyle.
